# The Role and Mechanism of ATM-Mediated Autophagy in the Transition From Hyper-Radiosensitivity to Induced Radioresistance in Lung Cancer Under Low-Dose Radiation

**DOI:** 10.3389/fcell.2021.650819

**Published:** 2021-05-12

**Authors:** Qiong Wang, Yangyang Chen, Haiyan Chang, Ting Hu, Jue Wang, Yuxiu Xie, Jing Cheng

**Affiliations:** Cancer Center, Union Hospital, Tongji Medical College, Huazhong University of Science and Technology, Wuhan, China

**Keywords:** low-dose radiation, HRS/IRR, autophagy, ATM, lung cancer

## Abstract

**Objective:** This study aimed to investigate the effect of ataxia telangiectasia mutated (ATM)–mediated autophagy on the radiosensitivity of lung cancer cells under low-dose radiation and to further investigate the role of ATM and its specific mechanism in the transition from hyper-radiosensitivity (HRS) to induced radioresistance (IRR).

**Methods:** The changes in the HRS/IRR phenomenon in A549 and H460 cells were verified by colony formation assay. Changes to ATM phosphorylation and cell autophagy in A549 and H460 cells under different low doses of radiation were examined by western blot, polymerase chain reaction (PCR), and electron microscopy. ATM expression was knocked down by short interfering RNA (siRNA) transfection, and ATM-regulated molecules related to autophagy pathways were screened by transcriptome sequencing analysis. The detection results were verified by PCR and western blot. The differential metabolites were screened by transcriptome sequencing and verified by colony formation assay and western blot. The nude mouse xenograft model was used to verify the results of the cell experiments.

**Results:** (1) A549 cells with high expression of ATM showed positive HRS/IRR, whereas H460 cells with low expression of ATM showed negative HRS/IRR. After the expression of ATM decreased, the HRS phenomenon in A549 cells increased, and the radiosensitivity of H460 cells also increased. This phenomenon was associated with the increase in the autophagy-related molecules phosphorylated c-Jun N-terminal kinase (p-JNK) and autophagy/Beclin 1 regulator 1 (AMBRA1). (2) DL-Norvaline, a product of carbon metabolism in cells, inhibited autophagy in A549 cells under low-dose radiation. DL-Norvaline increased the expression levels of ATM, JNK, and AMBRA1 in A549 cells. (3) Mouse experiments confirmed the regulatory role of ATM in autophagy and metabolism and its function in HRS/IRR.

**Conclusion:** ATM may influence autophagy through p-JNK and AMBRA1 to participate in the regulation of the HRS/IRR phenomenon. Autophagy interacts with the cellular carbon metabolite DL-Norvaline to participate in regulating the low-dose radiosensitivity of cells.

## Introduction

Radiotherapy is an important treatment method for lung cancer. Radiotherapy resistance and damage to surrounding normal tissue during radiotherapy limit its application in clinical practice. Some tumor cells exhibit hyper-radiosensitivity (HRS) to low-dose radiation: Within a radiation dose range < 1 Gy, cell destruction is enhanced per unit dose at an extremely low dose (<0.3–0.5 Gy), while cells show induced radioresistance (IRR) to higher doses (Marples and Collis, 2008). Due to the presence of the HRS/IRR phenomenon, at the same total dose, compared with conventionally fractionated radiotherapy, low-dose fractionated radiotherapy can significantly increase the radiosensitivity of tumor cells, increase the tumor control rate, and protect normal tissues (Valentini et al., 2010; Gupta et al., 2011; Dilworth et al., 2013; Schoenherr et al., 2013; Zhang et al., 2015). Few studies have focused on the mechanism of HRS in tumor cells, but whether this HRS phenomenon can be applied in conventional radiotherapy is an important topic.

Ataxia telangiectasia mutated (ATM) protein is an important factor affecting the radiosensitivity of tumor cells (Cremona and Behrens, 2013). When radiation causes DNA double-strand breaks (DNA DSBs), the Mre11–Rad50–Nbs1 complex first recognizes DSBs, then activates the kinase activity of ATM proteins and enables the phosphorylation of downstream substrates involved in DNA damage repair and cell cycle arrest, thereby playing a role in the recognition and repair of DSBs (Lavin et al., 2015). Xue et al. (2009, 2015). Observed the HRS/IRR phenomenon in human skin fibroblasts after carbon ion irradiation, and the HRS/IRR phenomenon showed different responses to treatment with specific ATM activators or inhibitors before radiation. Słonina et al. (2018) showed that in the fibroblasts of patients with HRS-positive tumors, the HRS phenomenon was closely related to the number of focal points of phosphorylated (p)-ATM. Enns et al. (2015) detected p-ATM expression in GM38, A549, and MCF7 cells after low-dose radiation. The results showed that the level of p-ATM was significantly increased in the presence of IRR. The above experimental results all suggest that ATM plays an important role in the transition from HRS to IRR under low-dose radiation.

Autophagy plays an important role in tumor radiotherapy. After autophagy is initiated, a series of phagy activities can be carried out through lysosomes to remove radiation-induced injury In lung cancer H1299 cells, radiotherapy-induced DNA damage regulates cellular autophagy through the ATM–MAPK14 pathway, thereby regulating the radiosensitivity of H1299 cells (Gewirtz, 2014; Liang et al., 2015). Our previous study showed that autophagy participated in the regulation of the transition from HRS to IRR in A549 lung cancer cells. HRS was enhanced in A549 cells by the autophagy inhibitor 3-methyladenine (3-MA) (Zhao et al., 2013). Therefore, we speculated that the transition from HRS to IRR, which involves autophagy, is regulated by the ATM gene, and the involved signaling pathways were explored in this study.

## Materials and Methods

### Cells and Radiotherapy

A549 and H460 human lung adenocarcinoma cell lines were purchased from the Cell Bank of Type Culture Collection of Chinese Academy of Sciences, Shanghai, China. They were cultured in RPMI 1640 medium at 37°C and 5% CO_2_. The concentration of fetal calf serum (FBS) was 10%; RPMI-1640 and FBS were purchased from Gibco (United States). After thawing, the cells did not contain mycoplasma. The number of aggregates (up to five cells) was kept as low as possible. Irradiation was performed using a Varian Unique-SN2236 linear accelerator (Siemens, Germany) at a dose rate of 1.32 cGy/s. Cellular radiosensitivity was detected using the colony formation method.

#### Colony Formation

Approximately 300 cells were inoculated into six-well plates and irradiated at the dose of 0, 0.2, 0.3, 0.5, 0.8, 1.0, or 2.0 Gy. The culture medium was replaced every 3–4 days. After incubation for 10–14 days, colonies were fixed with paraformaldehyde and stained with standard crystal violet solution. Colonies containing more than 50 cells were counted, and the plating efficiency was calculated as follows: (number of colonies/number of cells seeded) × 100%. Using the R language, the survivor fraction (SF) was fitted using the modified induced repair model (MIRM) with the following formula:

SF = exp(−αD −βD 2)

α = α_*res*_ {1 + [α_*sen*_/(α_*res*_−1)]} exp(−D/Dc)

The colony formation results were used for MIRM model construction in the R language. The survival curves were fitted, and the parameters were calculated: D = radiation dose value, α_*sen*_ = enhanced radiation effect at low-dose irradiation, α_*res*_ = radiation effect at high-dose irradiation, and Dc = dose inflection point at which HRS transforms to IRR. The standard for the existence of HRS/IRR is α_*sen*_ > α_*res*_, whose confidence limit has no overlap, along with Dc > 0.

### Western Blot

Total protein was extracted using a combination of RIPA buffer, phenylmethylsulfonyl fluoride, and phosphatase inhibitors. The protein lysates were separated by sodium dodecyl sulfate–polyacrylamide gel electrophoresis and then transferred to poly(vinylidene fluoride) membranes. The membranes were blocked in 5% skim milk at room temperature for 1 h and then incubated with the designated primary antibodies at 4°C overnight. The excess primary antibody was washed off with Tris-buffered saline with Tween 20, and the corresponding secondary antibody was incubated at room temperature for 1 h. A United Kingdom UV imaging system was used for exposure imaging. ImageJ was used to analyze the experimental results. Western Blot antibodies:ATM antibody, Cell signaling (#2873); Phospho-ATM (Ser1981) antibody, Cell signaling (#13050); LC3B antibody, Abcam (AB192890); SAPK/JNK Antibody, Cell signaling (#9252); Phospho-SAPK/JNK (Thr183/Tyr185) (81E11) antibody (#4668); AMBRA1 antibody, AB clonal (A12578); GAPDH antibody, AB clonal (AC002).

### Polymerase Chain Reaction (PCR)

After the cells were subjected to different treatments, cellular RNA was extracted on ice to prevent RNA degradation. The cells were evenly spread in the six well plates, and cell densities were adjusted as needed. The entire process was handled on ice. The medium was removed and the cells washed twice with PBS. Trizol (1 ml) was added and the cells were kept on ice for 5 min before being transferred to a 1.5-ml eppendorf tube. After chloroform (200 μl) was added to the tube, the mixture was vortexed until fully emulsified, and then centrifuged at 4°C for 15 min. About 200–400 μl of the aqueous phase was transferred to a new 1.5-ml tube. An equal volume of isopropanol was added and mixed well. The mixture was then centrifuged at 12,000 × *g* at 4°C for 10 min. The supernatant was discarded. The precipitate was dried for a few minutes and then dissolved in DEPC water. The concentration of RNA was measured. The optimal concentration was 300–500 ng/ml. 10-μl reverse transcription reactions were prepared. 500 ng RNA and 2-μl 5× prime script RT Master Mix buffer were added to each 10-μl reaction. The above system was prepared on ice, and then put in a polymerase chain reaction (PCR) machine for reverse transcription using the following reaction conditions: 37°C for 15 min; 85°C for 5 s; 4°C for 4 min. cDNA was stored at −80°C. The relative expression levels of target mRNA were calculated and statistically analyzed.

### Short Interfering RNA (siRNA) Transfection

Cells were digested, counted, and then cultured in six-well plate; the cell density on the next day was approximately 40–50%. Two RNase-free 1.5-ml Eppendorf tubes (A and B) were used, and 250 μl Opti-MEM + 5 μl RNAiMAX was added into tube A, while 250 μl Opti-MEM + 5 μl short interfering RNA (siRNA) was added into tube B. Opti-MEM was purchased from Gibco. The solutions were uniformly mixed by gently pipetting, then allowed to stand for 5 min. Solutions in tube A and tube B were uniformly mixed by gently pipetting and allowed to stand for 20 min. The mixed solutions of the two tubes were added into six-well plates and gently mixed. After 6 h, the medium was replaced with normal complete medium. The transfection efficiency was determined by PCR and western blot.

siATM1: CUGCCGUCAACUAGAACAUTT

AUGUUCUAGUUGACGGCAGTT

siATM2: GCAGUAUGCUGUUUGACUUTT

AAGUCAAACAGCAUACUGCTT

siATM3: GCUUGAGGCUGAUCCUUAUTT

AUAAGGAUCAGCCUCAAGCTT

siRNA JNK: 5″-AGAAGGUAGGACAUUCUUU-3″

siRNA AMBRA1: 5″-GGCCTATGGTACTAACAAA-3″

### Electron Microscopy

Electron microscopy is still one of the most accurate methods for quantitating autophagic vacuole accumulation. A549 and H460 cells were cultured in 6-well plates. The cells were subjected to varying doses of low radiation, and then collected in 2–4 h after radiotherapy by trypsinization and centrifugation at 1000 rpm for 5 min. The supernatant was discarded, and electron microscope fixatives were slowly added along the tube walls. Cells were then refrigerated at 4°C until used. The electron microscope specimens were sent to Wuhan Google Biotechnology Co. for subsequent treatment. The results of the electron microscopy were statistically analyzed.

### Transcriptome Sequencing

Short interfering RNA transfection knocked down the gene expression of ATM in A549 cells. After 48–72 h of transfection, the cells were collected, and 1 ml TRIzol was added to each well of a six-well plate. The cell suspension was transferred into a 1.5-ml Eppendorf tube and placed in a −80°C freezer for later use. The samples were sent to BGI Group, China for subsequent treatment. Kyoto Encyclopedia of Genes and Genomes (KEGG) pathway analysis was used to screen the genes related to autophagy. After obtaining the analytical results, the sequencing results were validated by PCR.

### Metabolomic Analysis

Short interfering RNA transfection knocked down the gene expression of ATM, JNK, and autophagy/Beclin 1 regulator 1 (AMBRA1) in A549 cells. The cells were scraped off using a sterile scraper, and then the cells were aspirated into the cryopreservation tube and centrifuged at 1000 rpm for 5 min. The supernatant was discarded, and the cryopreservation tube was quickly frozen in liquid nitrogen. The specimen was sent to Anachro Technologies Inc. for gas chromatography–mass spectrometry metabolomic sequencing. In the VIP scores graph, the larger the VIP value means the greater contribution. Generally, the variables with the VIP value greater than 1 have significant differences. In the *t*-test diagram, each colored dot represents a metabolite. The points marked in the graph are the metabolite with VIP value greater than 1 obtained by PLS-DA analysis.

### Nude Mouse Xenograft Model

One hundred microliters of cell suspension (1 × 10^8^ cells mixed with 50 μl Matrigel) was subcutaneously inoculated into 5-week-old female nude mice. When the tumor volume reached approximately 120 mm^3^, nude mice were randomly divided into six groups: control group, ATM inhibitor group, 2 Gy radiotherapy group, 0.2 Gy radiotherapy group, 2 Gy + ATM inhibitor group, and 0.2 Gy + ATM inhibitor group. BALB/C nude mice were used in the experiment, with five mice in each group. The concentration of ATM inhibitor (Ku-55933, Selleck, NO s1092) was 10 mg/kg. The method of administration was intraperitoneal injection. The drug was given 1 day before radiotherapy and every other day. In the process of radiotherapy, the interval of 0.2 Gy irradiation was 3 min. Tumor volume and mouse body weight were measured every 2–3 days. Animal experiments followed the *Guidelines for the Care and Use of Experimental Animals* (Ministry of Science and Technology of China, 2006) and were approved by the Animal Care and Use Committee of Tongji Medical College of Huazhong University of Science and Technology, China.

### Statistical Analysis

The experimental data were statistically analyzed using GraphPad Prism 5.0. One-way analysis of variance and Student’s *t*-test were performed. *P* < 0.05 indicated that a difference was statistically significant.

## Results

### The Sensitivity of Human Lung Cancer A549 and H460 Cells to Low-Dose Radiation and the Differences in ATM Expression

**FIGURE 1:**
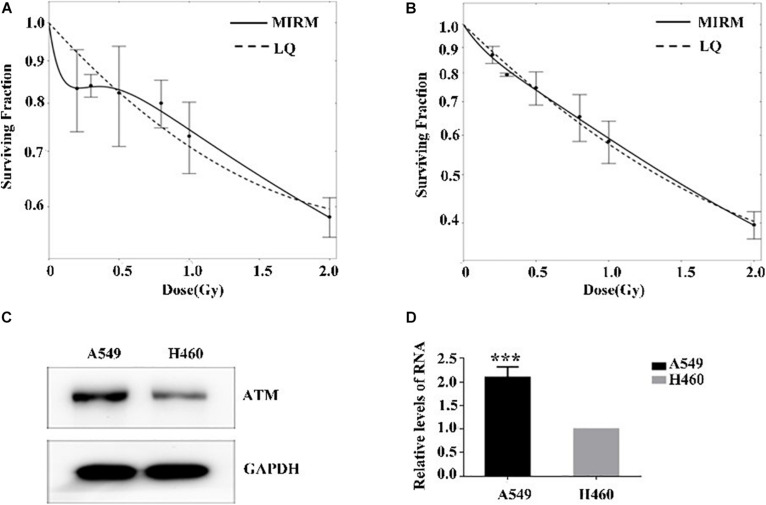
Low-dose radiosensitivity and ATM protein expression in A549 cells and H460 cells. **(A)** A549 cells showed radioresistance to 0.3–0.5 Gy. **(B)** The radiosensitivity of H460 cells was dose-dependent. **(C,D)** The mRNA and protein levels of ATM in A549 cells were both higher than those of H460 cells. *** *p* < 0.001.

The radiosensitivity of A549 and H460 cells exposed to low-dose irradiation was examined using clone survival analysis. Radioresistance was observed in A549 cells at 0.3–0.5 Gy, which is consistent with the HRS/IRR phenomenon. In addition, the radiosensitivity of H460 cells was dose-dependent, in line with the linear–quadratic (LQ) model (Figures 1A,B).

The ATM expression at the transcriptional and translational levels in human lung adenocarcinoma A549 and H460 cells were detected by PCR and western blot. In the HRS-positive A549 cells, the ATM mRNA and protein expression were higher than those of the H460-negative H460 cell line (Figures 1C,D).

### Correlation Between ATM and the HRS/IRR Phenomenon in A549 and H460 Cells

**FIGURE 2:**
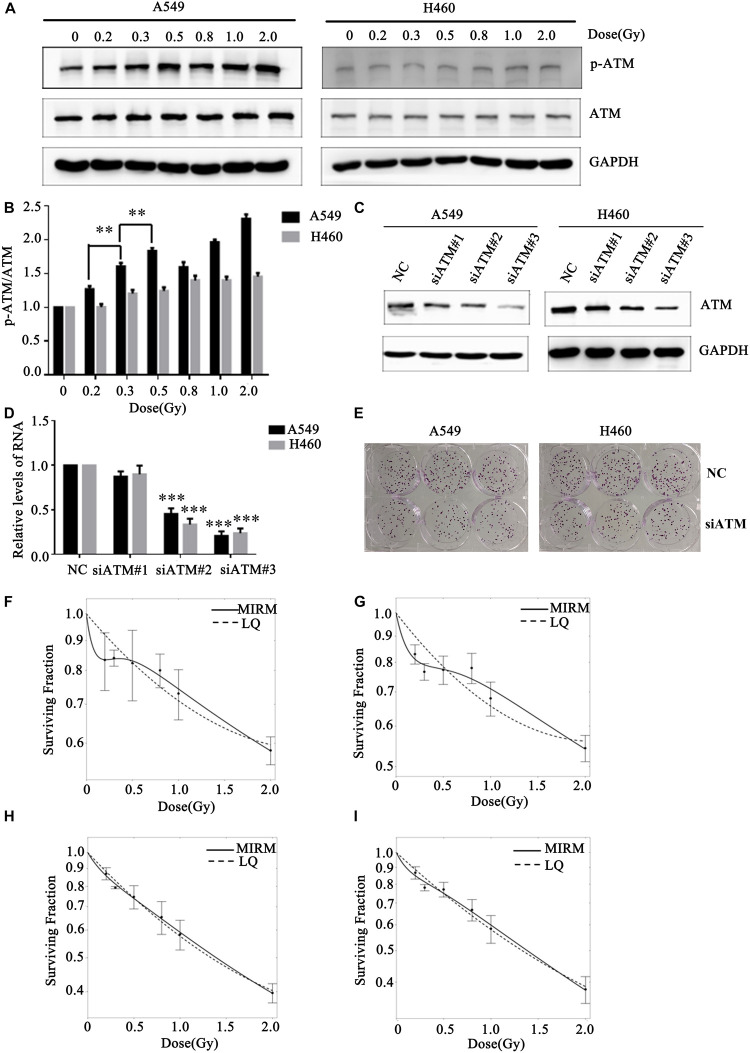
Effect of ATM on the low-dose radiosensitivity of A549 and H460 cells. **(A,B)** Level of p-ATM in A549 and H460 cells under low-dose radiation. **(C,D)** siRNA transfection knocked down ATM gene expression in A549 and H460 cells. **(E)** Decreasing the expression of ATM inhibited the proliferation of A549 and H460. **(F)** The survival curve of A549 cells fitted by MIRM showed the HRS/IRR phenomenon. **(G)** The HRS phenomenon of A549 cells was enhanced after ATM knockdown. **(H)** The survival curves of H460 cells were in line with the traditional LQ model. **(I)** Radiosensitivity was enhanced in H460 cells after ATM knockdown. *** *p*< 0.001, ** *p*< 0.01.

Human lung cancer A549 and H460 cells were irradiated with the dose of 0, 0.2, 0.3, 0.5, 0.8, 1.0, or 2.0 Gy. Western blot was used to detect changes in ATM phosphorylation level in the two groups of cells. After A549 cells received different low-dose radiotherapies, the p-ATM was upregulated and showed dose-dependent effects (in the case of low-dose irradiation). There was significant phosphorylation after 0.3 and 0.5 Gy irradiation, which was in line with a transition from HRS to IRR in A549 cells. The relative expression levels of p-ATM in H460 cells after 0.2, 0.3, and 0.5 Gy irradiation did not have statistically significant differences (Figures 2A,B).

To further confirm whether ATM participates in the regulation of the HRS/IRR phenomenon under low-dose radiation, we used siRNA transfection to downregulate the expression of ATM protein in A549 and H460 cells. We also used the colony formation method to analyze the changes in radiosensitivity at low doses in A549 and H460 cells. Compared to the negative control, siRNA2 and siRNA3 significantly reduced ATM expression in A549 and H460 cells (Figures 2C,D). Decreased expression of ATM makes the clone number of A549 and H460 cells decrease significantly (Figure 2E). The survival curve of A549 cells fitted by MIRM showed the HRS/IRR phenomenon (Figure 2F). After knockdown of ATM, the HRS phenomenon of A549 cells was enhanced (Figure 2G); the survival curve of H460 cells was consistent with the traditional LQ model (Figure 2H), and radiosensitivity was also increased at low doses in H460 cells after ATM downregulation (Figure 2I).

### ATM Regulates the Autophagy Levels in A549 Cells Under Different Low Doses of Radiation

**FIGURE 3:**
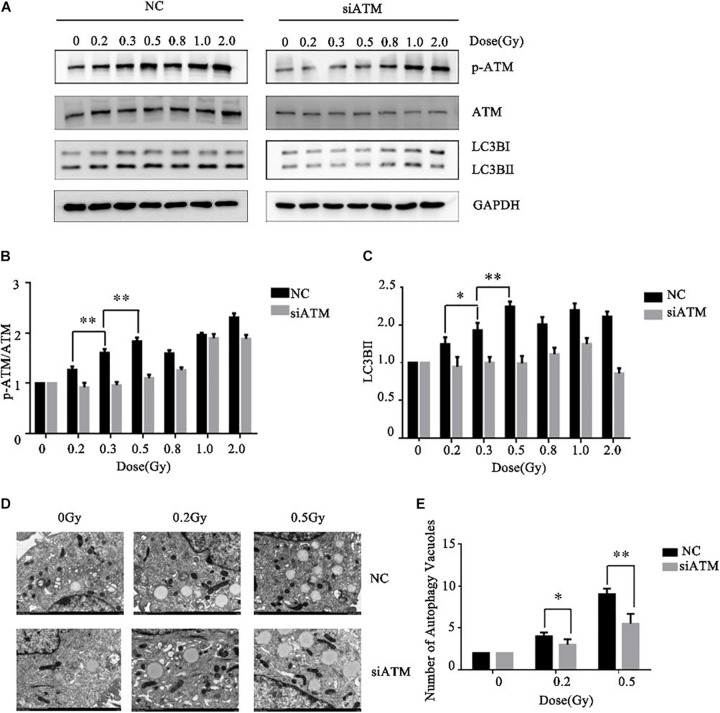
Ataxia telangiectasia mutated (ATM) regulates the level of autophagy in A549 cells after radiotherapy. **(A–C)** Changes in p-ATM and autophagy levels in the control group and ATM-knockdown group under low-dose radiation were detected by western blot. **(D,E)** Changes in the number of cellular autophagic vacuoles in the control group and ATM-knockdown group were detected by electron microscopy. ** *p*< 0.01, * *p*< 0.05.

Ataxia telangiectasia mutated protein expression in A549 cells was downregulated by siRNA transfection. The control group and ATM knockdown group were given low-dose irradiation of 0, 0.2, 0.3, 0.5, 0.8, 1.0, or 2.0 Gy. The effects of ATM knockdown on the phosphorylation level of ATM and the cellular autophagy level under different amounts radiation were analyzed by western blot. In the range of low-dose irradiation (≤0.5 Gy), the expression levels of p-ATM and LC3-II in the control group increased dose-dependently, and the increases were more significant at 0.3 and 0.5 Gy, while they decreased at 0.8 Gy. This manifestation was consistent with the transition from HRS to IRR in A549 cells. Knockdown of ATM inhibited ATM phosphorylation and the increase in cellular autophagy under low-dose radiation (Figures 3A–C).

To further confirm the above experimental results, we prepared cells 4–6 h after radiotherapy for electron microscopy. The results are shown in Figures 3D,E. ATM knockdown inhibited the increase in the number of autophagic vacuoles in A549 cells under low-dose radiation.

### Changes in ATM-Regulated Autophagy Pathway–Related Molecules Under Low-Dose Irradiation

**FIGURE 4:**
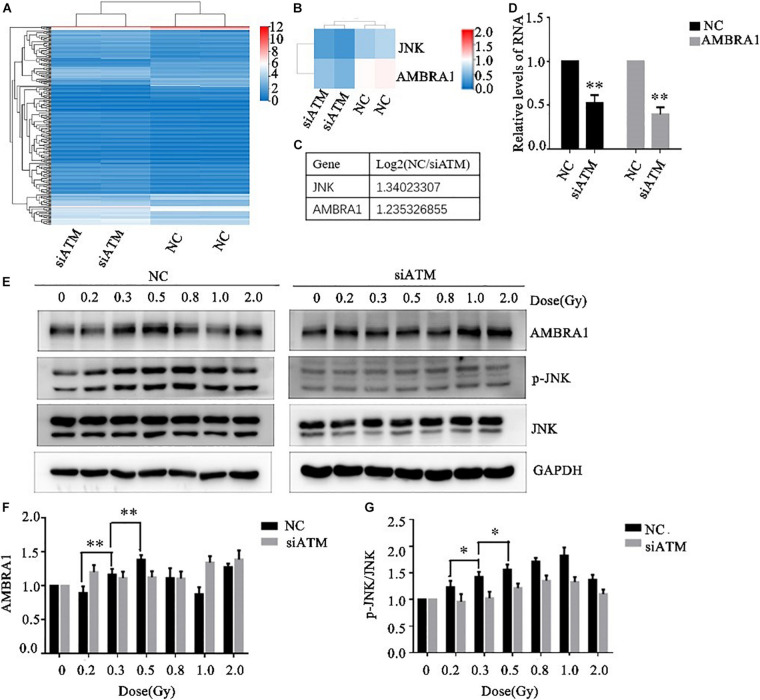
Ataxia telangiectasia mutated (ATM) regulates changes in autophagy pathway-related molecules. **(A–C)** Transcriptome sequencing analysis of the differences in autophagy pathway–related molecules between the control group and the ATM downregulated group. The differences in JNK and AMBRA1 between the two groups were the most significant. **(D)** The sequencing results were verified by PCR. **(E–G)** Changes in the levels of p-JNK and AMBRA1 in the control group and the ATM-knockdown group under low-dose radiation. ** *p*< 0.01, * *p*< 0.05.

The A549 cells in the control group and ATM knockdown group were subjected to transcriptome sequencing, and the molecules involved in the regulation of autophagy downstream of ATM were analyzed through KEGG pathway analysis. The differences in the expression of JNK and AMBRA1 were the most significant. In addition, the sequencing results were verified by PCR (Figures 4A–D).

Various low doses of radiation were given to the control group and the ATM knockdown group, and the differences in phosphorylated c-Jun N-terminal kinase (p-JNK), JNK, and AMBRA1 expression were analyzed by western blot. In the range of low-dose irradiation (≤0.5 Gy), p-JNK and AMBRA1 increased in a dose-dependent manner in the control group; the expression increased more significantly at 0.3 and 0.5 Gy, decreased at 0.8 Gy, and increased again at 1 Gy. Both p-JNK and AMBRA1 were downregulated under low-dose radiation after ATM knockdown (Figures 4E–G).

### Effects of AMBRA1 and JNK on the Low-Dose Radiosensitivity and Autophagy Level of A549 Cells

**FIGURE 5:**
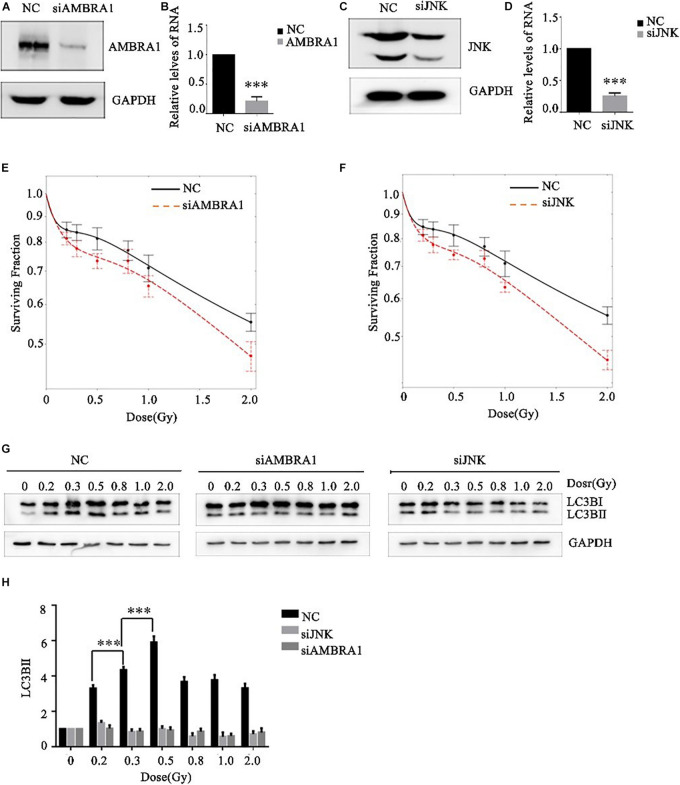
Effects of AMBRA1 and JNK on the radiosensitivity and autophagy of A549 cells. **(A,B)** siRNA transfection inhibited AMBRA1 gene expression in A549 cells. **(C,D)** siRNA transfection inhibited JNK gene expression in A549 cells. **(E,F)** When AMBRA1 and JNK were knocked down in A549 cells, the sensitivity of cells to low-dose radiation increased, and the HRS phenomenon was enhanced. **(G,H)** When AMBRA1 and JNK were knocked down in A549 cells, cellular autophagy under low-dose radiation was inhibited. *** *p*< 0.001.

JNK1 and AMBRA1 were knocked down in A549 cells by siRNA transfection, and the knockdown effect was verified by western blot and PCR (Figures 5A–D). Different (low) doses of radiation were given to the control group, JNK1-knockdown group, and AMBRA1-knockdown group. Colony formation analysis showed that knockdown of JNK1 and AMBRA1 affected the radiosensitivity of A549 cells at low doses. Specifically, it enhanced the low-dose radiosensitivity of A549 cells (Figures 5E,F).

After siRNA transfection knocked down JNK and AMBRA1 in A549 cells, western blotting was used to detect the expression of the autophagy-related molecule LC3B. Knockdown of JNK and AMBRA1 inhibited the increase in this autophagy protein at different low-dose radiation ranges (Figures 5G,H).

### Metabolic Processes Associated With Low-Dose HRS

**FIGURE 6:**
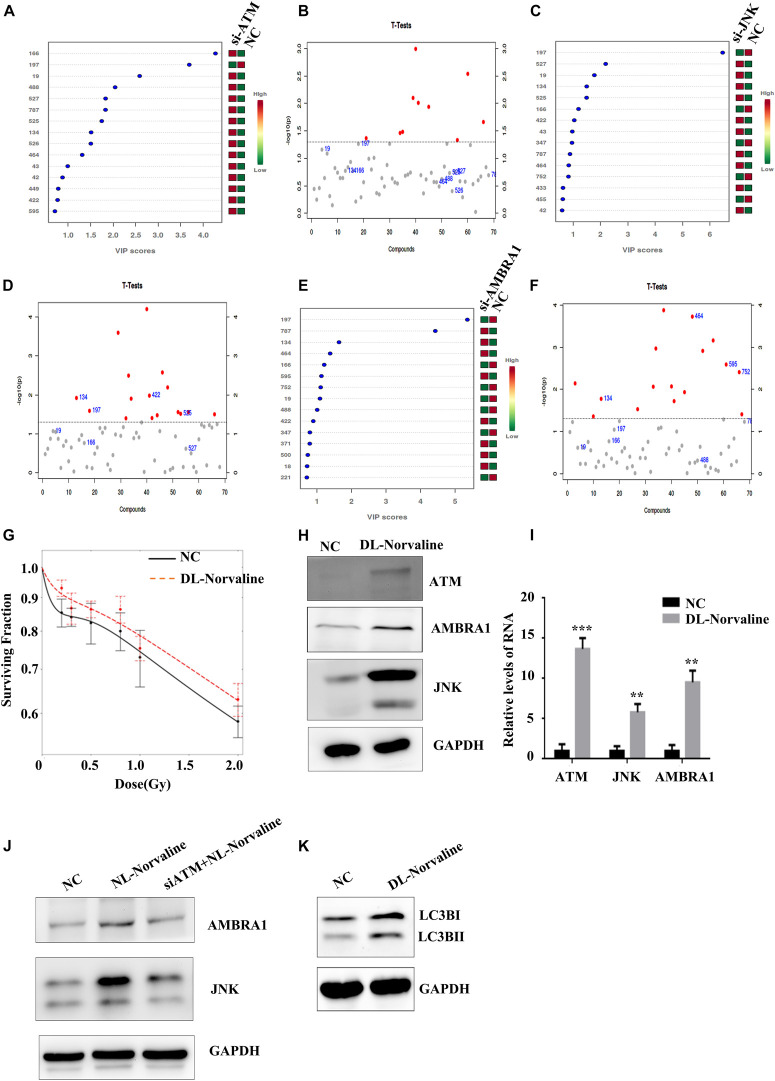
Metabolic processes associated with the low-dose HRS phenomenon. **(A,B)** Differences in metabolites between the ATM-knockdown group and the control group. **(C,D)** Differences in metabolites between the JNK-knockdown group and the control group. **(E,F)** Differences in metabolites between the AMBRA1-knockdown group and the control group. **(G)** Effect of DL-Norvaline on the low-dose radiosensitivity of A549 cells. **(H,I)** Effect of DL-Norvaline on the expression levels of ATM, JNK, and AMBRA1 in A549 cells. **(J)** Effect of siRNA knockdown of ATM on the DL-Norvaline effect. **(K)** Effect of DL-Norvaline on autophagy ***p < 0.001, **p < 0.01.

Cell metabolism plays an important role in tumor occurrence, development, and treatment (White et al., 2015). Cellular autophagy itself belongs to a process of catabolism, which is closely related to the metabolic processes of glucose metabolism, lipid metabolism, and amino acid metabolism in tumor cells, and these processes complement each other (Kimmelman and White, 2017). We screened the differential metabolite DL-Norvaline that is regulated jointly by ATM, JNK, and AMBRA1 through metabolomic sequencing (Figures 6A–F) and verified the effect of DL-Norvaline on A549 cell radiosensitivity at low doses through colony formation. The results showed that DL-Norvaline reduced the sensitivity of A549 cells under low-dose radiation (Figure 6G). We also examined the effect of DL-Norvaline (Selleck, art. No. s6334, the concentration was 10 ug/ml) on the expression levels of ATM, JNK, and AMBRA1 in A549 cells using western blot. The results showed that DL-Norvaline increased the expression levels of ATM, JNK, and AMBRA1 in A549 cells (Figures 6H,I). Next, we examined the expression of AMBRA1 and JNK in the negative control group, DL-Norvaline treatment group, and siATM + DL-Norvaline treatment group. The results showed that siRNA knockdown of ATM inhibited the increase in AMBRA1 and JNK expression by DL-Norvaline. Finally, we tested the effect of DL-Norvaline on autophagy level of A549 by Western blot. The results showed that DL-Norvaline increased the autophagy level of A549 cells (Figures 6J,K), which further confirmed the interaction within ATM, DL-Norvaline and autophagy.

### The Effect of ATM on Tumor Growth Under Low-Dose Radiation Is Verified by *in vivo* Experiments

**FIGURE 7:**
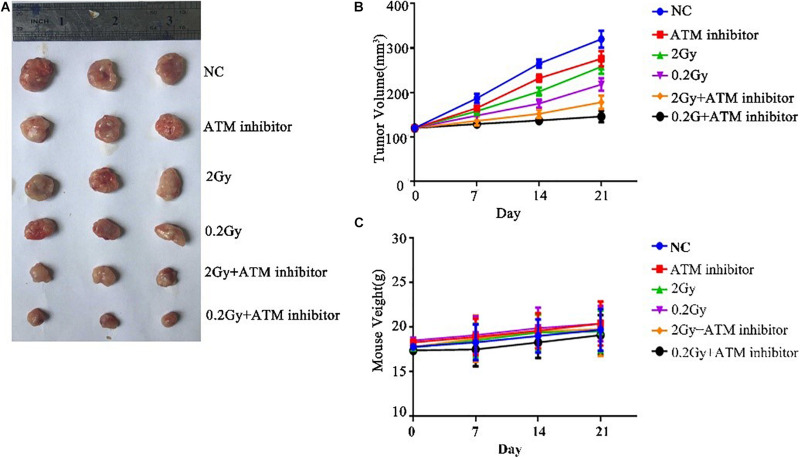
The effect of ATM on tumor growth under low-dose irradiation. **(A,B)** Effects of different treatment conditions on tumor xenograft volume in nude mice. **(C)** Effects of different treatment conditions on the body weight of nude mice.

To explore the effect of ATM on tumor growth under low-dose radiation, female nude mice were used as study objects. Nude mice were randomly divided into six groups: control group, ATM inhibitor group, 2 Gy radiotherapy group, 0.2 Gy radiotherapy group, 2 Gy + ATM inhibitor group, and 0.2 Gy + ATM inhibitor group. Compared with the negative control, the ATM inhibitor alone inhibited the growth of A549 tumors; 2 Gy/session × 5 sessions of radiotherapy alone or 0.2 Gy × 10/session × 5 sessions of radiotherapy alone slowed down the proliferation of A549 cell tumors. Compared to 2 Gy/session × 5 sessions of radiotherapy, 0.2 Gy × 10/session × 5 sessions had a more pronounced inhibitory effect on tumor growth. When radiotherapy and ATM inhibitors were used jointly, 0.2 Gy × 10/session × 5 sessions had a more pronounced inhibitory effect on tumor growth (Figures 7A,B). There is no obvious difference within 6 treatment conditions on the body weight of nude mice (Figure 7C).

## Discussion

The low-dose ultra-microfractionated radiotherapy technique divides the conventional dose into several parts and delivers them at a certain intervals (Miciak and Bunz, 2017; Terashima et al., 2017). Due to the presence of low-dose HRS, hyper-fractionated low-dose radiotherapy has unique clinical advantages. However, the current research on the mechanism of low-dose HRS is still not very clear (Olobatuyi et al., 2017, 2018).

This study used HRS/IRR-positive A549 cells and HRS/IRR-negative H460 cells as study objects. The expression level of ATM in HRS-positive A549 cells was significantly higher than that in HRS/IRR-negative H460 cells. After different low-dose radiation treatments of A549 cells, p-ATM was significantly upregulated in a dose-dependent manner, most significantly after 0.3 and 0.5 Gy, whereas the relative expression levels of p-ATM in H460 cells were not significantly different between the 0.2, 0.3, and 0.5 Gy groups. After siRNA knockdown of ATM, the sensitivities of A549 cells and H460 cells under low-dose radiation were both increased, and the HRS of A549 cells was enhanced. The above experimental results all confirm that ATM was involved in the regulation of low-dose radiation hypersensitivity in lung cancer cells.

Our previous experiments showed that treatment with the autophagy inhibitor 3-MA enhanced radiosensitivity under low doses in A549 cells, suggesting that autophagy is an important factor inducing HRS/IRR phenomenon (Zhao et al., 2013). Did ATM-regulated autophagy participate in the regulation of the sensitivity of the A549 lung cancer cells at low doses and participate in the transition from HRS to IRR? Our results showed that in the low-dose range (≤0.5 Gy), the expression levels of p-ATM and LC3-II in the control group increased in a dose-dependent manner, most significantly after 0.3 and 0.5 Gy. After siRNA knockdown of ATM, the phosphorylation of ATM and the increase in autophagy levels under low-dose radiation were inhibited. Our electron microscopy results also showed that after inhibiting the expression of ATM, the number of autophagic vacuoles in A549 cells decreased, and the volume of autophagic vacuoles relatively increased under different low doses of radiation. These results further confirm our hypothesis that ATM-regulated autophagy is involved in the transition from HRS to IRR under low-dose radiation.

To further investigate the mechanism through which ATM regulates autophagy under low-dose radiation and thereby regulates the transition of HRS to IRR, we screened for differentially expressed genes associated with autophagy pathways downstream of ATMs by transcriptome sequencing analysis. This screen returned JNK and AMBRA1. JNK, the c-Jun N-terminal kinase, is a member of the mitogen-activated protein kinase family and belongs to protein kinases. After JNK is activated, it regulates cell proliferation, survival, differentiation, and metabolism through downstream substrates (Wu et al., 2019). JNK is involved in the autophagy induced by various types of stimuli, including nutritional deficiency, reduction of cytokines and growth factors, and neurotoxic drugs (Vegliante et al., 2016; Zhang et al., 2018; Bai et al., 2019). JNK activation can induce Bcl-2 phosphorylation, destroy the Bcl-2/Beclin 1 complex, and promote the release of Beclin 1 to induce autophagy (Yang et al., 2015). AMBRA1, also known as autophagy/Beclin-1 modulator 1, plays an important role in the regulation of autophagy and acts as a positive regulator of Beclin-1, thereby promoting the formation of autophagosomes (Capizzi et al., 2017). Our results showed that under low-dose radiation (≤0.5 Gy), the levels of p-JNK and AMBRA1 in A549 cells increased in a dose-dependent manner; the increases were most significant at 0.5 Gy; and ATM knockdown inhibited the increases in p-JNK and AMBRA1 after different low doses of radiation. The above experimental results confirm that ATM can regulate JNK phosphorylation and AMBRA1 expression under low-dose radiation.

To further verify whether ATM regulated the autophagy level of cells under low-dose radiation through JNK1 and AMBRA1 and thereby regulate the radiosensitivity of A549 cells, we knocked down JNK1 and AMBRA1 in A549 cells through siRNA transfection and analyzed the effects of JNK1 and AMBRA1 on the radiosensitivity of A549 cells under low doses by a colony formation assay. The results showed that JNK1 and AMBRA1 knockdown increased the radiosensitivity of A549 cells at low doses. At the same time, we examined the autophagy levels of cells under different low doses of radiation. JNK1 and AMBRA1 knockdown inhibited the increase in autophagy levels in A549 cells caused by low-dose irradiation. The above experimental results further confirmed that ATM might regulate the radiosensitivity of A549 cells under low-dose irradiation through p-JNK and AMBRA1.

Autophagy is inseparable from many cellular metabolic processes. We screened the differential metabolite DL-Norvaline by metabolomic analysis. DL-Norvaline is an important metabolic product involved in the process of carbon metabolism in cells. The colony formation assay showed that DL-Norvaline inhibited the autophagy level of A549 cells under low-dose radiation. DL-Norvaline increased the expression levels of ATM, JNK, and AMBRA1 in A549 cells. siRNA knockdown of ATM inhibited the increased expression of AMBRA1 and JNK by DL-Norvaline. Therefore, we hypothesized that under low-dose radiation, ATM interacts with DL-Norvaline through autophagy regulated by JNK and AMBRA1 to jointly regulate the low-dose radiosensitivity of A549 cells. Finally, we validated the results of cell experiments in mouse experiments.

## Conclusion

In conclusion, our study found that ATM may affect autophagy through p-JNK and AMBRA1 and thus participate in the regulation of HRS/IRR phenomenon. Autophagy participates in the regulation of low-dose radiosensitivity in cells by interacting with DL-Norvaline, a carbon metabolism product. Next, we will validate and investigate the regulatory mechanism of this HRS in conventional-dose and high-dose radiotherapy to help improve the radiosensitivity of lung adenocarcinoma.

## Data Availability Statement

The original contributions presented in the study are publicly available. This data can be found here: https://www.ncbi.nlm.nih.gov/bioproject/PRJNA725505, PRJNA725505.

## Ethics Statement

The animal study was reviewed and approved by Huazhong University of Science and Technology Institutional Animal Care and Use Committee.

## Author Contributions

QW: conceptualization, investigation, data curation, formal analysis, methodology, validation, visualization, and writing–original draft. YC: conceptualization, investigation, data curation, methodology, and visualization. HC: investigation and re-sources. TH: investigation. JW and YX: methodology and resources. JC: conceptualization, data curation, formal analysis, funding acquisition, supervision, validation, visualization, and writing–review and editing. All authors contributed to the article and approved the submitted version.

## Conflict of Interest

The authors declare that the research was conducted in the absence of any commercial or financial relationships that could be construed as a potential conflict of interest.
